# Preparation and Performance Evaluation of a Plugging Agent with an Interpenetrating Polymer Network

**DOI:** 10.3390/gels9030205

**Published:** 2023-03-07

**Authors:** Zengbao Wang, Yitian Liu, Weian Huang, Xiong Yang, Ziao Liu, Xushuo Zhang

**Affiliations:** 1 School of Petroleum Engineering, China University of Petroleum (East China), Qingdao 266580, China; 2 Key Laboratory of Unconventional Oil & Gas Development, China University of Petroleum (East China), Ministry of Education, Qingdao 266580, China

**Keywords:** interpenetrating polymer network, elastic particle plugging agent, viscoelasticity, temperature resistance, plugging performance

## Abstract

In view of the problems of polymer cross-linked elastic particle plugging agents commonly used in oilfields, including easy shear, poor temperature resistance, and weak plugging strength for large pores, the introduction of particles with certain rigidity and network structure, and cross-linking with a polymer monomer can improve the structural stability, temperature resistance, and plugging effect, and the preparation method is simple and low-cost. An interpenetrating polymer network (IPN) gel was prepared in a stepwise manner. The conditions of IPN synthesis were optimized. The IPN gel micromorphology was analyzed by SEM and the viscoelasticity, temperature resistance, and plugging performance were also evaluated. The optimal polymerization conditions included a temperature of 60 °C, a monomer concentration of 10.0–15.0%, a cross-linker concentration of 1.0–2.0% of monomer content, and a first network concentration of 20%. The IPN showed good fusion degree with no phase separation, which was the prerequisite for the formation of high-strength IPN, whereas particle aggregates reduced the strength. The IPN had better cross-linking strength and structural stability, with a 20–70% increase in the elastic modulus and a 25% increase in temperature resistance. It showed better plugging ability and erosion resistance, with the plugging rate reaching 98.9%. The stability of the plugging pressure after erosion was 3.8 times that of a conventional PAM-gel plugging agent. The IPN plugging agent improved the structural stability, temperature resistance, and plugging effect of the plugging agent. This paper provides a new method for improving the performance of a plugging agent in an oilfield.

## 1. Introduction

Profile control and water plugging technologies have always been effective means to improve the injection of water and achieve stable production of oil from reservoirs in oilfields. An elastic plugging agent has outstanding characteristics of being able to “enter, block, and move”, which helps in realizing deep water plugging and profile regulation, increasing the ripple volume, and improving water flooding. It also plays a very important role in water-plugging profiles, such as pre-cross-linked water expansion [1,2,3] and colloidal dispersion of gel particles [4,5,6], nanospheres [7,8,9], etc. However, after the elastic particles absorb water and expand, they can be easily sheared and broken during the formation and migration processes, which affects the plugging strength, especially for large pores with ultra-high permeability and fractured oil reservoirs, which makes it difficult to achieve effective plugging. In addition, most of its temperature resistance fails to meet the requirements of medium- and high-temperature formations, which limits its application scope.

An interpenetrating polymer network (IPN) gel is a novel gel consisting of two or more polymer networks intertwined into a structure [10,11,12]. Compared with traditional polymer gels, IPN gels have a special network structure and there is synergy between the polymer chains, which imparts the gel with higher shear resistance, plugging ability, profile improvement, and erosion resistance [13]. The commonly used methods for preparing IPN are as follows:

(1) Homogeneous interpenetrating liquid systems: Aalaie [14,15] prepared a semi-IPN gel using a homogeneous liquid system with polyacrylamide as the first network, followed by mixing with an aqueous polyvinyl alcohol solution or an aqueous solution of hard dextran. This system was mainly researched for water plugging in an oil well. Liu Yongbing [16,17] evenly mixed polyvinyl alcohol, acrylamide, acrylic acid, and an initiator and copolymerized them to obtain an IPN gel for deep oilfield flooding, which showed enhanced oil recovery. Luo Yi [18] synthesized an organic–inorganic gel system with an IPN structure based on sol-gel technology, using water-soluble phenolic resin, water glass, and a water inhibitor as materials, which meets the requirements of deep plugging and could be used as a temperature-resistant plugging agent.

(2) Interpenetrating emulsion systems: Yang Xiufen [19] used a W/O (water-in-oil) emulsion system of polyacrylamide (TDGIR), a cross-linking agent, modified amino resin (TF-3), and a curing agent to prepare an IPN gel profile modulator (TDG-IR/TF-3), which was suitable for profile adjustment of water injection wells at 35–150 °C. Zhao Xiulan [20] prepared an IPN gel, TDG, from latex with a W/O/W (water-oil-water) structure and modified amino resin at a certain temperature using a cross-linking agent and a coagulant. Liu Qingpu [21] cross-linked W/O polyacrylamide latex with modified amino resin in the presence of a cross-linking agent to obtain a cross-penetrating polymer network gel as a water plugging agent for selective water plugging in oil wells.

(3) Interpenetrating solid-phase systems: Ma Tao [22] prepared a two-component IPN/montmorillonite composite hydrogel using a composite monomer, natural polymeric material, montmorillonite, a cross-linker, and an initiator. It was used to adjust the water absorption profile of the water injection well and direct the in-depth flow of liquid. Yan Yonggang [23] added sodium-based soil into the chromium gel to enable its interpenetration into the spatial network structure of partially hydrolyzed polyacrylamide (HPAM) gel and obtained a high-strength IPN gel. Shen Qun [24] developed an IPN gel system suitable for the reservoir by using a polymer, fly ash, cross-linking agent A, and cross-linking agent B for the liquid profiling of a low-temperature and low-permeability fractured sandstone reservoir. It had an improved cross-section and showed selective plugging performance by plugging the water layer and not blocking the oil layer.

In recent years, there has been less research interest on IPN gels, mainly due to the underground gumming of IPN gels that are greatly affected by the salinity of the formation. Moreover, high temperature formation also affects the gumming process, which is limited during the actual process. Compared with the gel system, the IPN particles are less affected by the salinity of the formation, have the advantages of high blocking strength and ease of synthesis and storage, and are more convenient for practical applications.

The polymer introduced by Tang Xiaofen [25] could control the rate of water absorption into the expanded network structure of a conventional water absorbent. The prepared IPN gel system displayed excellent characteristics, such as delayed expansion and high strength. Liu Lijun [26] introduced modified polyvinyl alcohol into the polymerization system containing AA-AM monomers and prepared polyacrylic acid-absorbent resin by polymerization in aqueous medium. The relationship between the particle size of the absorbent resin and the water absorption rate was then studied. Yang Fan [27] blended polyvinyl alcohol (PVA) with other polymers to prepare two different superabsorbent resins, poly(acrylic-acrylamide) (PAA-AM) and a semi-interpenetrating network of polyvinyl alcohol/poly(acrylic-acrylamide) (PVA/PAA-AM). However, these studies are not aimed at oilfield profile control and water plugging. Chen Xing [28] prepared acrylamide-based composite cross-linked microspheres of different particle sizes by reversed-phase fine emulsion and reversed-phase suspension polymerization methods using acrylamide and 2-acrylamide-2-methylpropanesulfonic acid (AMPS) as monomers, a stable cross-linker, and an unstable cross-linker. Their anti-swelling characteristics and control methods, as well as microsphere hydrogel toughening methods, were discussed. The study was based on oilfield profile control and water plugging, but was not applied in the field.

Compared with the traditional profile control agent for controlling water plugging, the IPN plugging agent has a unique network structure. The synergy between the polymer chains can improve the performance of the plugging agent. Thus, they can meet the needs of profile control and water plugging in the oilfield and have good development potentials. However, the IPN gels currently used as plugging agents are mainly homogeneous elastic particles, which are easy to break by shear during long-distance transport in the formation and have a short plugging period. Generally, the preparation method and process are relatively complex and the cost is high, which limits its widespread application. The IPN gel prepared in this study takes particles with certain rigidity and a network structure as the first network, and cross-links and interpenetrates with the polymeric monomer to form a structure with “inner rigidity and outer flexibility”, which improves the structural stability, temperature resistance, and plugging effect of the plugging agent, and the preparation method is simple and low-cost.

## 2. Results and Discussion

### 2.1. Optimization of Preparation Conditions

#### 2.1.1. Polymerization Temperature

Maintaining the dissolution temperature of 30 °C and the concentration of acrylamide monomer as 10.0 wt%, the polymerization temperature was chosen based on the morphologies obtained at different polymerization temperatures. The experimental results are shown in Table 1.

**Figure 1:**
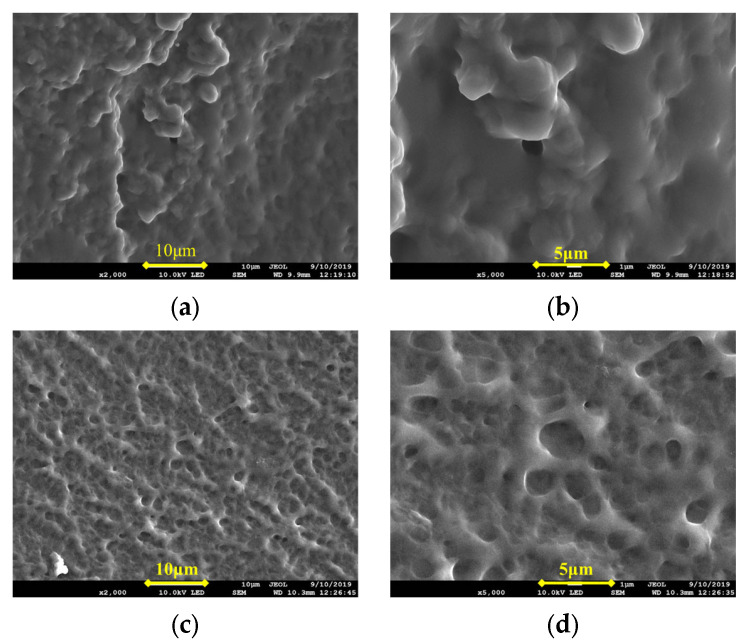
SEM images of frozen colloids prepared at reaction temperatures of 60 °C and 80 °C. (**a**) 60 °C ×2000; (**b**) 60 °C ×5000; (**c**) 80 °C ×2000; and (**d**) 80 °C ×5000.

As can be seen from Table 1, with the increase of temperature, the reaction to the formation of elastic colloid was shorter. At a lower temperature, the monomer molecules moved at a low speed, the rates of chain initiation and chain growth were lower, and the polymerization reaction took a longer time. As the temperature increased, the monomer molecules moved at an accelerated speed. Along with it, the chain initiation and chain growth rates were also accelerated, and the time required for a polymerization reaction was shortened. As the temperature continued to rise, the number of active centers increased, and the chances of collisions increased due to the heat released during the reaction increasing, and the viscosity of the aqueous solution increasing, which makes the heat difficult to emit, resulting in the phenomenon of flash polymerization. As a result, the chains broke easily, and the length of the main chain decreased. Finally, the molecular weight and degree of polymerization decreased. In Figure 1, the network structure of the frozen colloid prepared at 80 °C appeared to be looser than that prepared at 60 °C. There were a larger number of holes in the network, and the skeletal structure was relatively loose. As a result, the frozen colloid prepared at 80 °C showed certain brittleness at a macro level, and its strength was lower than that at 60 °C. The optimum polymerization temperature was found to be 60 °C.

#### 2.1.2. Monomer Concentration

Maintaining a dissolution temperature of 30 °C, a reaction temperature of 60 °C, and a reaction time of 4 h, the amount of the monomer was varied. The optimal monomer concentration was determined on the basis of the morphologies of the reaction products. The experimental results are presented in Table 2.

As can be seen from Table 2, with the increase of the concentration of the polymerization monomer, the cross-linked product changed from viscous fluid to elastic gels. When the concentration exceeds 20%, the reaction is violent, resulting in violent flash polymerization. With a low monomer concentration, the probability of contact between monomers and collisions was low, which was not conducive for the growth of molecular chains. This made the polymerization difficult due to the low reaction rate, and the reaction took a longer time for completion. As the monomer concentration increased, the probability of collisions between the monomers increased, the molecular chain length increased, and the reaction speed was accelerated. However, when the monomer concentration was too high, the probability of molecular collisions was very high, and the reaction rate increased rapidly, which was accompanied by a release of heat. Thus, the rise in temperature of the system accelerated the rate of the reaction, resulting in heat dissipation from the reaction system and, consequently, flash polymerization. This was not favorable for improvement of the molecular weight of the polymer. The results show that the optimal concentration of the monomer was 10.0–15.0%, hence, a monomer concentration of 10.0% was chosen for further experiments in this study.

#### 2.1.3. Dosage of Cross-linking Agent

Maintaining a dissolution temperature of 30 °C, reaction temperature of 60 °C, a reaction time of 4 h, and a monomer concentration of 10.0%, the concentration of the cross-linking agent was varied. On the basis of the morphology of reaction products and the reaction process, the amount of the cross-linking agent was decided. The experimental results are presented in Table 3.

From Table 3, we determined that increasing the concentration of the cross-linking agent can improve the strength of the cross-linked product, but if the concentration exceeds 3.0%, it will lead to flash polymerization. The optimal concentration of the cross-linking agent N, N’-methylene diacrylamide was found to be 1.0–2.0% of the monomer concentration. In this paper, the concentration of the cross-linking agent was chosen as 1.0% of the monomer concentration.

**Figure 2:**
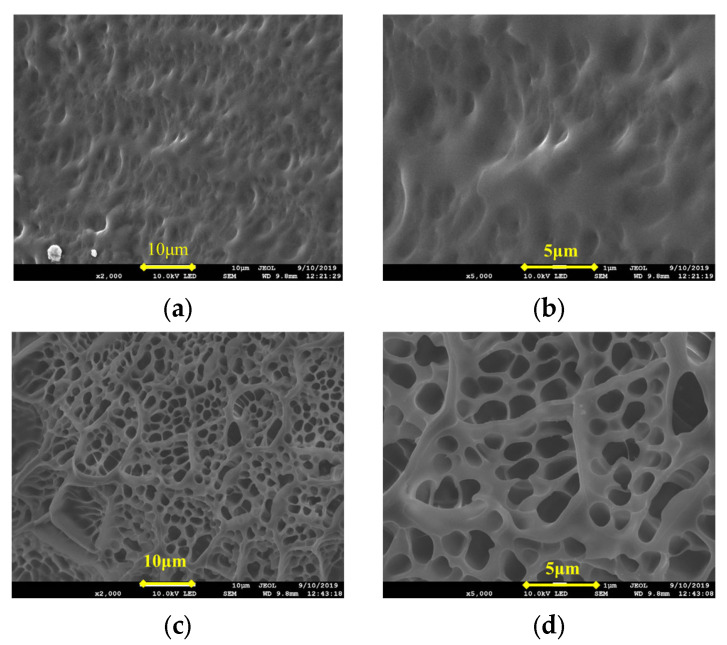
SEM images of the frozen colloids prepared with 1.0% and 3.0% concentrations of the cross-linking agent. (**a**) 1.0%, ×2000; (**b**) 1.0%, ×5000; (**c**) 3.0%, ×2000; and (**d**) 3.0%, ×5000.

At a low concentration of the cross-linking agent, N, N’-methylene diacrylamide, the allyl groups at both ends of N, N’-methylene diacrylamide molecule were less likely to open up and simultaneously cross-link with the monomer, and the cross-linking was not obvious. As the concentration of the cross-linking agent increased, the cross-linking reaction between the cross-linking agent and the monomer increased, and the product was obtained as a frozen colloid. The microstructures of the frozen colloid in Figure 2a,b revealed that at 1.0% concentration of the cross-linking agent, the product appeared dense with a tight skeleton and long vertical and horizontal network chains, and the product had the characteristics of high strength, toughness, and elasticity. As the concentration of the cross-linking agent continued to increase, the rate of the reaction accelerated, accompanied by release of a large amount of heat. The temperature of the system rose, and the reaction speed was further accelerated, resulting in flash polymerization. The microstructures in Figure 2c,d show that the frozen colloid structures were relatively loose, with an obvious “honeycomb briquette-like” structure. The monomers were only polymerized and showed circular rings with short molecular chains. The product characteristics included low strength and high brittleness and were prone to breakage.

#### 2.1.4. First Network Load

Maintaining a dissolution temperature of 30 °C, a reaction temperature of 60 °C, a reaction time of 4 h, an acrylamide monomer concentration of 10.0%, and a cross-linking agent concentration of 1.0%, the concentration of the first network particle was varied, and the experimental results are shown in Table 4.

Homogeneously cross-linked polymeric gels could be obtained using the first network particles in a certain concentration range. When the particle concentration exceeded 25% of the monomer concentration, the distance between the particles decreased, the electrostatic force and van der Waals force increased, and the agglomeration between the particles occurred easily, allowing for phase separation to occur in polymerized cross-linked gel. To achieve the product at a low cost and with high strength of the plugging agent, a higher concentration of the first network produced better reaction products on the premise that the reaction products do not appear in phase separation. The overall results in Table 4 show that the optimal concentration of the first network was 20.0% of the concentration of the monomer.

### 2.2. Microstructure

**Figure 3:**
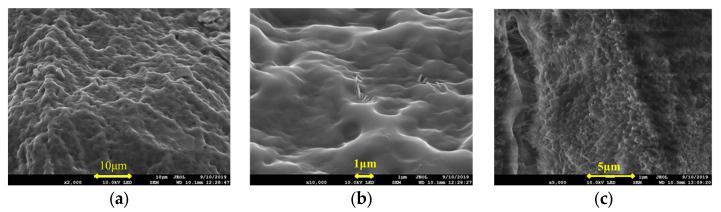

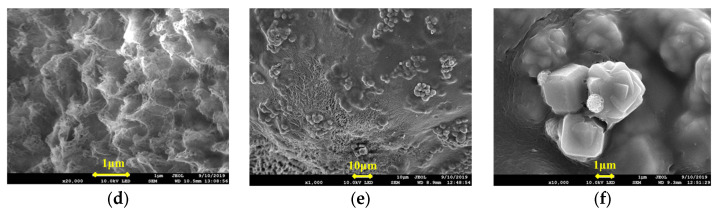
SEM Images of (**a**) @sericite, ×2000; (**b**) @sericite, ×10,000; (**c**) @lithium saponite, ×5000; (**d**) @lithium saponite, ×20,000; (**e**) @ zeolite powder, ×1000; and (**f**) @ zeolite powder, ×10,000.

The microstructures of the IPNs prepared using lithium saponite, sericite, and zeolite powder particles with the first network are shown in Figure 3.

In Figure 3a,b, the skeleton of the cross-linked IPN gel @sericite appears dense with evenly distributed sericite particles as flakes and filaments. The sericite particles were not agglomerated, and their flake and filamentous particles were randomly spread in two dimensions. The particles were evenly interspersed and disordered, which increased the strength of the frozen colloid. When the cross-linked gel was stretched or compressed, cracks appeared that expanded vertically and encountered the sericite particles, after which the crack propagation ceased. In case of further crack propagation, it was necessary to destroy the bridging effect of the sericite in the cross-linked gel. Failure of this bridging action would lead to one of the following two cases: structural fracture of the sericite or the dissociation of the sericite from the polymeric colloid. Since the structural strength of the sericite was higher than that of the cross-linked matrix, the main action involved in the destruction of the cross-linking was the cleavage of the hydrogen bonding between the fixed sericite and its dissociation from the cross-linked gel matrix. When the dissociation reached a certain extent, the cross-linked gel was broken. Due to the lamellar and filamentous structure of sericite in the frozen colloid, the separation of sericite from the colloid and the destruction of hydrogen bonds during the fracturing of the cross-linking gel were not in the same plane of crack propagation. The main crack in the fractured cross-linked gel turned in the direction along the sericite shape, which led to an increase in the propagation path, increasing the surface area of the crack, and finally highly increased resistance to propagation [29,30]. This directly manifested as an increase in the strength of IPN.

The SEM images of the IPN@ lithium saponite cross-linked gel structure (Figure 3c,d) showed that they were similar to that of acrylamide cross-linking gel as a whole, with “gully and streak”-shaped veins. In contrast to the structure of cross-linked acrylamide gel, lithium saponite showed a dioctahedral structure with layered silicate, with special properties, such as water absorption, high expansion, additional electrolytic action, and action of organic gum [31]. After the expansion of the lithium saponite due to water absorption, the acrylamide monomers entered into the structure and blended themselves during the cross-linking. Due to the interpenetration and blending of the lithium saponite, small protrusions were seen in the shapes of “gully” and “stripe”. This blend showed a multi-dimensional orientation that increased the strength of the IPN.

In Figure 3e,f, the powdered particles of the zeolite and polyacrylamide cross-linking gel do not show close combination and no substantial interpenetrating network structure was formed. Most of the powdered particles of zeolite were found to remain “free” outside the cross-linked polyacrylamide structure with obvious phase separation. Summarily, the powdered zeolite particles were present as aggregates.

The reason for this could be due to the porous framework of the zeolite powder, which was small and had high surface energy. When encountering water, water absorption will lead to binding between the particles, the distance between the particles will be shortened, the intermolecular force and electrostatic force will increase, and agglomeration of mutual attraction between particles occurs. The power of mechanical stirring during the synthesis could not disperse them completely, so they were coated onto the polyacrylamide gels. The coated aggregates of the zeolite powder were cross-linked with acrylamide through hydrogen bonding. Due to agglomeration, it was difficult for the aqueous acrylamide solution to enter into the aggregates and the interpenetration of the particles failed, and thus showed obvious phase separation.

**Figure 4:**
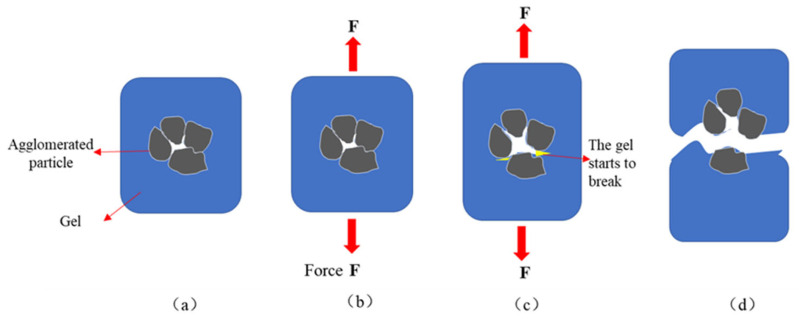
Schematic diagram of tensile fracture process in IPN@zeolite powder cross-linked gel. (**a**) The agglomerated particles are coated by gel; (**b**) The gel is pulled; (**c**) The gel began to break from the agglomerated particles; (**d**) The gel is stretched and broken.

The agglomeration of the particles reduced the toughness and plastic strength of the cross-linked gel. When the cross-linked colloid was compressed and stretched, the applied force was transferred to the aggregate. The chemical bond energies from the acrylamide cross-linking and the hydrogen bonding between the outside of the aggregate and the polymer were higher than the electrostatic and van der Waals forces between the aggregate particles. The aggregate was first pressed open or torn and the stress operation point was formed, which made the frozen colloid more readily prone to crushing or tearing. In other words, it had reduced toughness and plastic strength. Figure 4 shows the tensile fracture process of the interpenetrating network polymerization with zeolite powder.

Figure 4a shows the polymerization of the interpenetrating network with zeolite powder. Figure 4b shows the effect of applied tensile forces on the cross-linked gels. Figure 4c shows that, on increasing the tensile force, the electrostatic and van der Waals forces between the particles of the powdered zeolite aggregates were eliminated, and the cross-linked gel started tearing due to stress concentration. Figure 4d shows the torn state of the cross-linked gel.

By comparing the microstructural morphologies and strengths of the IPNs, the following conclusions could be drawn: (1) IPNs with a good degree of fusion and no phase separation could form multidimensional network structures with high strength. (2) Interpenetrating particles could increase the strength of IPNs by embedding themselves into the polymer skeleton, thus increasing the phase stripping resistance and blending with the polymer network structure. (3) The aggregation of particles could reduce the strength of IPNs.

### 2.3. Viscoelasticity

Viscoelasticity is a comprehensive property including material viscosity and elasticity, which reflects the solid and liquid properties of materials and is determined by measuring the storage modulus G’ and the loss modulus G” of the material. The storage modulus G’ (also known as the modulus of elasticity) represents the strength of the elasticity of the material, whereas the loss modulus G” (also known as the viscous modulus) characterizes the viscosity of the material.

**Figure 5:**
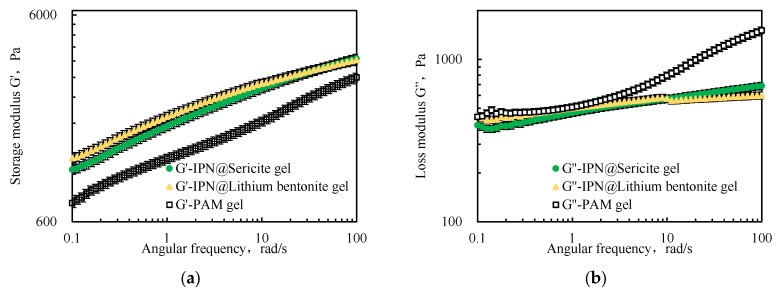
Comparison of (**a**) storage modulus G’ and (**b**) loss modulus G” of interpenetrating polymer networks.

It is evident from Figure 5 that, as the angular frequency increased, both the storage modulus G’ and the loss modulus G” increased, indicating that the conventional polyacrylamide cross-linked gel (PAM-gel) and the interpenetrating network polymeric cross-linked gel (IPN-gel) possessed viscoelastic structures. Moreover, the G’ value was always higher than the G” value, which indicates that the elastic properties of the IPN played a dominant role.

The G’ of the IPN@Particles-gel was higher than the G’ of the conventional PAM-gel. The low-frequency G’ value reflected the cross-linking strength of the elastomer material. The G’ of the IPN@Particles-gel was 1.2–1.7 times that of the PAM-gel based on G’ at an angular frequency of 0.1 rad/s, which reflected a better cross-linking strength.

The G” values of the IPN@Particles-gel were relatively concentrated and were basically similar to the G” values of the PAM-gel at low frequencies, because its viscosity characteristics were derived from the conventional polyacrylamide cross-linked gel. As the angular frequency was increased, the G” of the PAM-gel increased significantly, indicating a decrease in its stability. This further reflected that the structural stability of polymeric cross-linked gels was enhanced after the interpenetration of particles within the network structure.

### 2.4. Thermal Resistance

With developments and exploitation of potential oil and gas reservoirs under harsh conditions, the temperature resistance of plugging agents has become an important factor. The temperature resistance of IPNs was evaluated on the basis of water absorption expansion multiple and viscoelasticity. IPN@sericite-gel was selected for temperature resistance evaluation and compared with the conventional polyacrylamide cross-linked gel body PAM-gel.

**Figure 6:**
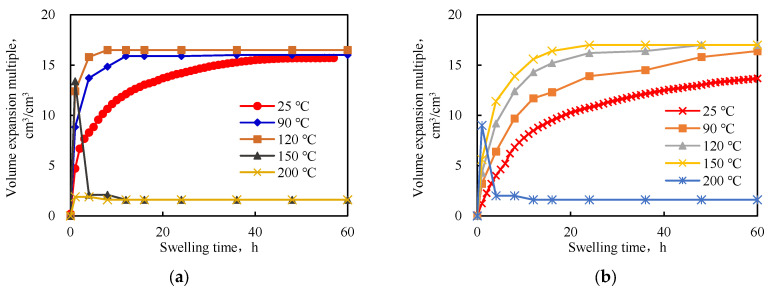
Curves for the swelling rates of the interpenetrating network aggregates at different temperatures: (**a**) PAM-gel; (**b**) IPN@sericite-gel.

From Figure 6, it is evident that as the temperature increased, the swelling rate accelerated and the equilibrium water absorption rate increased. This was due to the fact that as the temperature increased, the molecular movements increased, which increased the probability of rapid entry of the water molecules into the interpenetrating network space and the swelling rate was accelerated. With an increase in temperature, the equilibrium swelling ratio of the IPN@sericite-gel increased to a value higher than that of the PAM-gel. The analysis concluded that as the temperature increased, the hydrolysis of the acylamino groups into carboxylic acid groups on the polymerized cross-linked gel increased. Thus, the hydrophilicity of the polymer chain increased, and, consequently, the swelling factor increased. At the same time, with an increase in temperature, the conformational transition energy of the polymer chain of the interpenetrating network polymer increased, the physical entanglements between the molecular chains decreased to a certain extent, the degree of chain relaxation increased, and the water-absorbing expansion of the sample was reduced. This allowed more water to enter the interpenetrating network structure and the expansion multiple increased. The sericite network structure in the IPN@sericite-gel was less affected by temperature; no network relaxation occurred, the temperature rose, the water absorption expansion multiple did not change much, and the increased water absorption expansion multiple was mainly attributed to the hydrolysis of amide groups in the PAM-gel into carboxylic acid groups.

**Figure 7:**
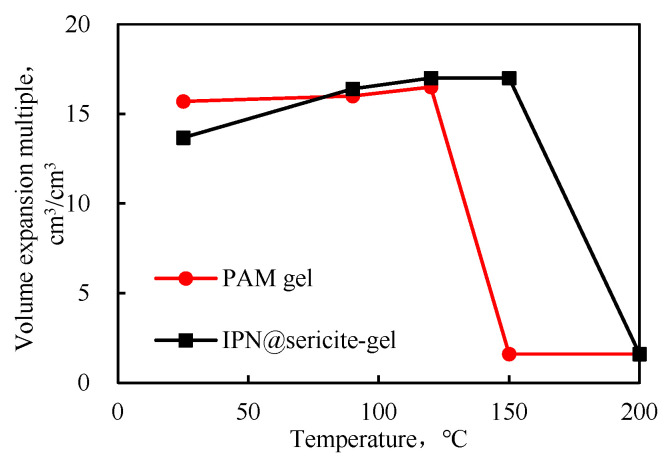
Equilibrium swelling factor at different temperatures.

The equilibrium swelling ratios of the two cross-linked gels at different temperatures are shown in Figure 7. As the temperature increased, the swelling ratio increased at first and then decreased. As mentioned earlier, the decrease in swelling was mainly due to the rupture of the polymer chains and the destruction of the interpenetrating network structure at a high temperature.

The performance of the PAM-gel changed significantly above 120 °C, and at 150 °C, the swelling ratio was low. The high-temperature aging form was found to be a viscous fluid, with the loss of characteristics of the granular plugging agent. The characteristics of the IPN@sericite-gel after aging at 200 °C were similar to those of the PAM-gel at 150 °C. Compared with the PAM-gel, the temperature resistance of the IPN@sericite-gel was improved from 120 °C to 150 °C by 25%.

**Figure 8:**
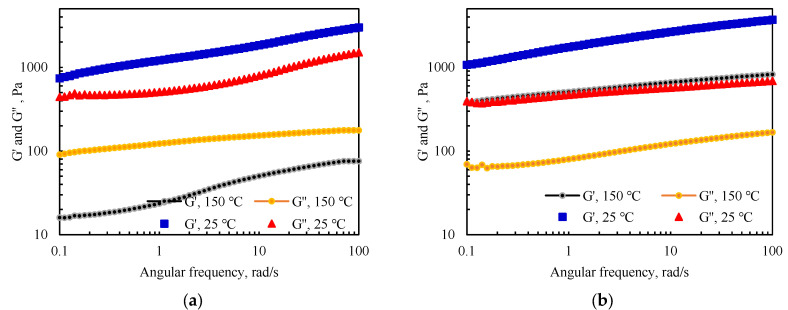
Comparison of the viscoelastic properties of the PAM-gel and the IPN@sericite-gel at 150 °C and 25 °C: (**a**) PAM-gel; (**b**) IPN@sericite-gel.

Based on the results of the water absorption expansion temperature at different temperatures, the viscoelasticity of the material after water absorption and swelling at 150 °C was tested. The fixed shear modulus was 0.5%, and the testing time was fixed at 10 s with an angular frequency of 0.1–100 rad/s. The experimental results were compared with the viscoelasticity model of the sample after water swelling at 25 °C and the results are presented in Figure 8.

After high-temperature aging and expansion, the energy storage modulus G’ and energy dissipation modulus G” of the PAM-gel and the IPN@sericite-gel decreased, which occurred due to the breaking of some polymer chains and the destruction of the IPN structures at high temperatures. After the aging of the PAM-gel at 150 °C, a change from G” < G’ to G” > G’ occurred at 25 °C. This indicated that the solid-state characteristics of the PAM-gel changed to those of liquid after high temperature aging, viscosity was prominent, and the cross-linked spatial network structure was destroyed into a polymeric chain structure. The IPN@sericite-gel still showed G’ > G” after aging at 150 °C and still exhibited elasticity of the solid, and the skeletal support of the frozen colloidal was still stable.

### 2.5. Plugging Performance

The sealing pressure of the hyperpermeable layer of the formation plugging and erosion resistance after plugging are important indicators for the evaluation of continuous effectiveness of the plugging agent in the water-plugging profile. The erosion resistance of the prepared particles of the IPN@sericite-gel plugging agent was evaluated and compared with the plugging performance and erosion resistance of the granular PAM-gel plugging agent and polymer solution. The experimental results are shown in Table 5 and Figure 8.

It is evident from Table 5 that the HPAM solution could achieve a good blocking rate with difficulty. The granular plugging agent prepared from the PAM-gel and the IPN@sericite-gel displayed a good and stable plugging rate of >95.0%. Amongst the plugging agents prepared, the IPN@sericite-gel showed the best stable plugging performance, with the plugging rate reaching 98.9%.

**Figure 9:**
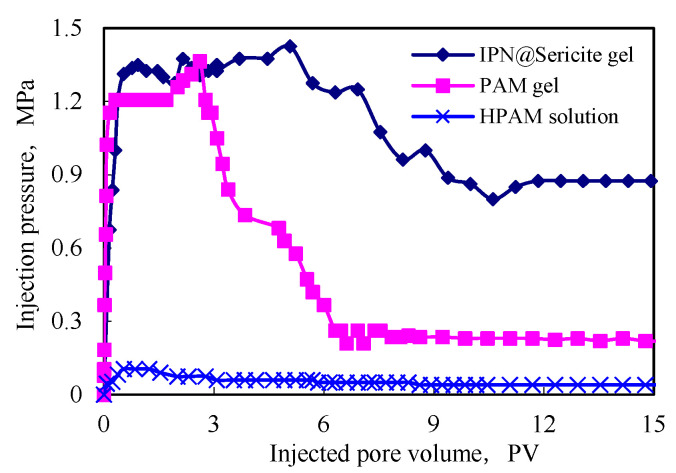
Comparison of blocking performances.

It is evident from Figure 9 that the injection pressure of the granular plugging agent was significantly higher than that of the HPAM solution, demonstrating its good plugging effect. The maximum pressure reached by using a granular plugging agent was basically the same. The maximum value of the pressure reflected the pressure of the flexible particles for starting the deformation. The transfer of the maximum pressure of the plugging agent prepared using the IPN@sericite-gel was slightly higher, indicating that the force required for the same deformation of the particles was also greater; that is, the strength was greater. The plugging pressure of the plugging agent began to decline after reaching the maximum pressure for some time. This was due to the shear of the particles of the plugging agent during the transfer process that caused the shear and crushing of the plugging agent particles, and then the plugging pressure began to gradually decrease.

The longer the duration of the maximum plugging pressure to maintain the scouring time, the better was the shear resistance of the plugging agent particles. The shear resistance in the formation was proportional to the erosion resistance time. After the scouring period, the plugging pressure reached a stable value, which indicated that after the transfer of shear, the particles formed a stable bridging structure in the formation. The particles could no longer move, and the pressure remained stable. It is evident from the figure that the maximum plugging pressure of the IPN@sericite-gel plugging agent had the longest erosion resistance time and the highest plugging pressure after washing, which was significantly better than that of the PAM-gel plugging agent. The stable plugging pressure was 3.8 times that of the PAM-gel plugging agent.

The value of the pressure stability reflected the stability and effectiveness of the plugging agent. During the migration process, the PAM-gel plugging agent was severely sheared and broken and the stable pressure value was small. Owing to the structure of the IPN@sericite-gel plugging agent, the PAM cross-linked gel encountered sericite during the shear and fragmentation processes, which hindered its further shearing. The sericite particles possessed high strength, and after the shear failure of the cross-linked gel, the sericite particles could still achieve secondary plugging. Due to this comprehensive action, the stability of the pressure of the IPN@sericite-gel plugging agent was higher than that of the PAM-gel plugging agent. This indicated that the IPN structure improved the plugging performance and erosion resistance of the plugging agent and achieved a better plugging effect.

## 3. Conclusions

(1) The IPN gel prepared takes particles with certain rigidity and network structure as the first network, and cross-links and interpenetrates with the polymeric monomer to form the structure of “inner rigidity and outer flexibility”, which can improve the structural stability, temperature resistance, and plugging effect of the plugging agent. The G’ value increased by 20–70%, the temperature resistance increased by 25%, the plugging rate reached 98.9%, and the stable plugging pressure after scouring was 3.8 times of that of the conventional PAM-gel plugging agent.

(2) Good integration between interpenetrating networks and the absence of phase separation were the prerequisites for the formation of high-strength interpenetrating network polymers. Aggregation of the particles also reduced the strength of the interpenetrating network. Therefore, a particle dispersant can be added to reduce the agglomeration problem.

(3) The cross-linked gel prepared by interpenetration of particles with a certain network structure has a simple preparation method and low cost. This study provides a new method for improving the performance of an oilfield plugging agent and has a good application prospect.

## 4. Materials and Methods

### 4.1. Instruments and Materials

#### 4.1.1. Instruments

A rheometer (MCR301, Anton Paar Co., Ltd., Graz, Austria) was used for testing the rheological properties of the polymers. The micromorphologies of the samples were studied using a field emission scanning electron microscope (SU8020, Hitachi Limited, Tokyo, Japan). A 101A-1E vacuum constant temperature drying oven, a pulverizer, a three-mouth flask, a serpentine condenser tube, etc. were used for the experiments.

#### 4.1.2. Materials

Acrylamide, sodium bisulfite, ammonium persulfate, sodium hydroxide, and N, N’-methylene bisacrylamide are chemically pure reagents that were purchased from Sinopharm Chemical Reagent Co. Ltd., China. Nitrogen, >99% purity; 10# white oil; 4A zeolite powder; lithium soap clay; sericite; and industrial products were purchased from Shandong Usolf Chemical Technology Co., Ltd., China.

### 4.2. Experimental Methods

#### 4.2.1. Synthesis Method

**Figure 10:**
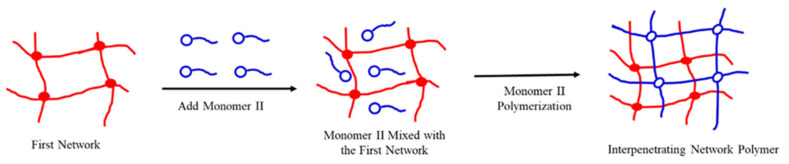
The diagram of interpenetrating polymer networks prepared by stepwise manner.

The interpenetrating polymer networks were prepared in a stepwise manner. Particles with network structure were introduced into another monomer or liquid oligomer system containing catalysts, cross-linkers, etc. After adsorption saturation, the monomer or oligomer was polymerized in-situ and cross-linked to form a second network [32]. The reaction scheme is shown in Figure 10.

The first network was zeolite powder, lithium saponite, or sericite particles with a network structure, and the second network was a polymerized monomer acrylamide and the cross-linker N, N’-methylene bisacrylamide. We dissolved N, N’-methylene bisacrylamide with 20 g of water, then added the first network particles into the solution, stirred evenly, and left the mixture for adsorption for 24 h. Then we added acrylamide and water to 100 g, and injected nitrogen into the solution for 20 min under a stirring state. Under the protection of nitrogen, the temperature was raised to the polymerization temperature, and the initiator system of ammonium persulfate and sodium bisulfite was added. During the synthesis, the first network released the cross-linker (concentration diffusion), whereas the second network interpenetrated into the first network. The reaction was stirred to the end of polymerization (forming a non-mobile elastic gel). Based on previous experiences of preparation [33] and literature procedures [34,35,36], the dissolution was conducted at 25–40 °C and the polymerization was conducted at 60–80 °C. The initiator system was comprised of ammonium persulfate and a sodium bisulfite composite in a molar ratio of 1:1, and the dosage accounted for 1.0% of the total monomer concentration.

#### 4.2.2. Viscoelastic Evaluation

The storage modulus G’ and loss modulus G” of the interpenetrating network aggregates were determined by rheological analysis. The fixed shear modulus was 0.5%, the test time was fixed as 10 s, and the angular frequency was 0.1–100 rad/s.

#### 4.2.3. Temperature Resistance Evaluation

The temperature resistance of the IPN was evaluated in terms of the expansion multiple during water absorption and viscoelasticity. The drainage volume method was used to test the expansion multiple and the rate of water absorption. The details of this method are as follows: a suitable measuring cylinder was chosen and labeled as 1 and filled with deionized water to half its volume capacity; the accurate volume was considered *V*_10_. Then a certain mass of IPN particle plugging agent was added into the graduated cylinder and the volume *V*_11_ at this time was quickly recorded. Since the volume of the IPN particle plugging agent increased after water absorption and expansion, the water in the graduated cylinder decreased. However, the expansion in volume could not be accurately read in the same graduated cylinder. We prepared another measuring cylinder 2, deionized water was added, and the volume was recorded as *V*_20_. The deionized water volume ensured that the subsequent water expansion could be submerged. Then, the expanded particles in graduated cylinder 1 were filtered and poured into graduated cylinder 2. The volume of graduated cylinder 2 was recorded as *V*_21_ at this time. This procedure was repeated by recording and calculating the expansion multiple at different expansion times. The water absorption expansion multiple *n* was calculated as follows:

$$n=\frac{V_{11}-V_{10}}{V_{21}-V_{20}}$$

A water bath was used for maintaining the temperature of less than 95 °C. For the water absorption swelling ratio experiments exceeding 100 °C, the IPN particles were transferred into an aging steel pipe containing sufficient boiling water (95–100 °C), sealed, kept in an oven at a certain temperature, taken out at regular intervals, washed with tap water to cool down, and the expansion multiple was measured.

#### 4.2.4. Plugging Performance

**Figure 11:**
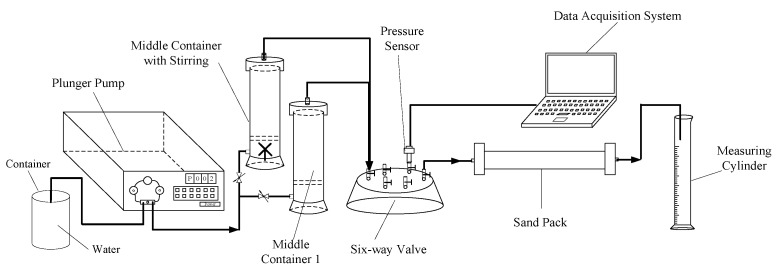
Comparison of blocking performances.

The sealing pressure of hyperpermeable layer of the plugging formation and the erosion resistance after plugging are important indicators for the evaluation of the continuous effectiveness of the plugging agent in the water-plugging profile. The prepared cross-linked gel was sheared and crushed, dried, and granulated. The plugging performance was evaluated by the sand-filled pipe displacement model, and the flow chart is shown in Figure 11. The sand-filled pipe used for the experiment was 30 cm long and had an inside diameter of 2.5 cm. It was filled with 60–80 mesh quartz sand. The particle size of the plugging agent was sieved into the sand-filled pipe using a 120-mesh standard sieve and then weighed. After saturation, the weight was measured and the pore volume of the simulated core of the sand-filled pipe was calculated based on the mass difference before and after saturation. Then a 1.0 PV plugging agent system (granular plugging agent concentration was 1.0%, HPAM solution was 3000 mg/L) was injected. After the plugging agent system was injected, water flooding was carried out, the injection pressure was recorded, and the stable plugging rate was calculated.

## Figures and Tables

**Table 1:** Reaction forms at different polymerization temperatures.

Polymerization Temperature, °C	Polymerization Modality	Reaction Phenomenon
40.0	Large elastic frozen colloids, good toughness	The complete reaction time was relatively longer, about 8–10 h.
60.0	Large elastic frozen colloids, good toughness	The reaction time was moderate, and the complete polymerization took about 2–4 h.
80.0	Large elastic frozen colloids showing certain brittleness, frozen colloids could be vigorously crushed	A sudden flash polymerization occurred within 1 h.

**Table 2:** Reaction phenomena under different amounts of polymerization monomers.

Monomer Concentration, %	Polymerization Modality	Reaction Phenomenon
3.0	Almost non-viscous aqueous solution	No obvious reaction with a low degree of polymerization
5.0	Viscous fluid with some fluidity	As reaction progressed, the viscosity of the system gradually increased
10.0	Large elastic frozen colloid, good toughness	The viscosity of the system increased and finally cross-linked to form a frozen colloid.
15.0	Large elastic frozen colloid, good toughness	The viscosity of the system increased quickly and finally, it cross-linked into a frozen colloid.
20.0	White frozen colloid, high elasticity	Flash polymerization
25.0	White frozen colloid, high elasticity	Flash polymerization
30.0	Frozen colloid, elastic, slightly brittle	Flash polymerization

**Table 3:** Reaction forms of different concentrations of cross-linkers.

Cross-linking Agent Concentration (Compared to the Polymeric Monomer), %	Polymerization Modality	Reaction Phenomenon
0.5	Viscous fluid	The viscosity of the system increased gradually and there was no cross-linking.
1.0	Frozen colloid with high elasticity, good toughness	The viscosity of the system increased at first, and then formed gels.
2.0	Frozen colloid with high elasticity, better toughness	The viscosity of the system increased at first, and then formed a gel, which reacted faster.
3.0	Frozen colloid with high elasticity, brittle and fragile	After reacting for a while, flash polymerization occurred.

**Table 4:** Morphologies of the interpenetrating polymer networks prepared using different concentrations of the first network particle.

First Network Concentration (Compared to the Polymeric Monomer) (%)	Polymerization Modality
5.0	The first network distribution was uniform, and the elasticity and toughness of the polymer frozen colloid were good.
10.0	The first network was evenly distributed, and the elasticity and toughness of the polymer frozen colloid were good.
15.0	The first network was evenly distributed, and the elasticity and toughness of the cross-linked gel were good.
20.0	The first network was evenly distributed, and the elasticity and toughness of the cross-linked gel were good.
25.0	The distribution of the first network was more uniform, and there was a slightly greater number of particles at the bottom of the reaction medium. The elasticity and toughness of the cross-linked gel were worse than those of the previous groups.
30.0	The distribution of the first network was more uniform, and there were slightly greater numbers of particles at the bottom of the reaction medium. The elasticity and toughness of the cross-linked gel worsened.

**Table 5:** Experimental results of erosion resistance of water expansion.

Plugging Agent System	Sand Filling Permeability K_1_, 10^−3^ mm^2^	Scouring Stable Permeability K_2_, 10^−3^μm^2^	Plugging Rate, %
IPN@sericite gel	1230.2	14.1	98.9
PAM gel	1098.6	49.9	95.5
HPAM solution	1155.6	288.9	75.0

## Data Availability

The data presented in this study are available upon request from the corresponding author.
